# Heat application in live cell imaging

**DOI:** 10.1002/2211-5463.13912

**Published:** 2024-11-03

**Authors:** Linda Sistemich, Simon Ebbinghaus

**Affiliations:** ^1^ Chair of Biophysical Chemistry Ruhr‐University Bochum Germany; ^2^ Research Center Chemical Sciences and Sustainability, Research Alliance Ruhr Bochum Germany

**Keywords:** fluorescence microscopy, live‐cell imaging, microheating, thermometry

## Abstract

Thermal heating of biological samples allows to reversibly manipulate cellular processes with high temporal and spatial resolution. Manifold heating techniques in combination with live‐cell imaging were developed, commonly tailored to customized applications. They include Peltier elements and microfluidics for homogenous sample heating as well as infrared lasers and radiation absorption by nanostructures for spot heating. A prerequisite of all techniques is that the induced temperature changes are measured precisely which can be the main challenge considering subcellular structures or multicellular organisms as target regions. This article discusses heating and temperature sensing techniques for live‐cell imaging, leading to future applications in cell biology.

## Graphical abstract

Thermal modulation in live‐cell imaging with a high spatiotemporal resolution allows the investigation of biomolecular reactions like protein deactivation and unfolding, and cellular processes like calcium signaling. Local changes in temperature are induced via infrared (IR) irradiation of water (direct), magnetic or radiative excitation of absorber material (indirect), or by stage heating and they can be monitored by optical thermometry.

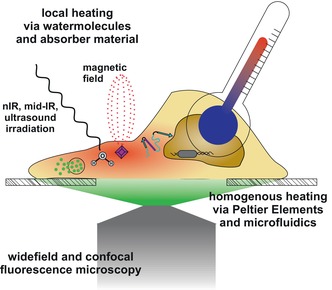

**Abbreviations**
FLIRT: fast local IR thermogenetics
FLUCS: focused light‐induced cytoplasmic streaming
FReI: fast relaxation imaging
FRET: Förster resonance energy transfer
GFP: green fluorescent protein
HIFU: high‐intensity focused ultrasound heating
Hsp: heat shock protein
IR: infrared
mid‐IR: middle infrared
MIP: mid‐infrared photothermal imaging
nanoHT: nanoheater‐thermometer
nIR: near infrared
PAPS: 3′‐Phosphoadenosin‐5′‐phosphosulfat
SOD1: superoxide dismutase 1
SPIONS: superparamagnetic iron oxide nanoparticles
TOOL: temperature oscillation optical lock‐in
TRP: transient receptor potential
TRPV: transient receptor potential vanilloid
ts: temperature sensitive
VIS: visible

Organisms of any complexity exist preferentially at their comfort temperature. Deviations from this temperature on a shorter or longer timescale lead to an adaption of the organism or degeneration and death at extreme conditions. Joel Asaph Allen noted in the 19th century, that animals living in cold climate regions show smaller extremities compared to their relatives living in warmer regions (Allen's law). While Allen's law is valid on an evolutionary timescale, astounding temperature effects are observed at short timescales, for example concerning the sex determination of the American alligator. Incubating the eggs in the 2 °C‐window between 32 °C and 34 °C results in a dramatically higher probability of male offspring, while temperatures below and above this range rather lead to females [1]. The understanding of such phenotypes requires studies on a cellular and molecular level.

The first question is, how different cells and organisms sense temperature on a molecular level. Biomacromolecules in the cell such as nucleic acids, lipids and proteins can indeed serve as temperature sensors. In pro‐ and eucaryotes, temperature‐dependent gene expression is controlled via DNA and RNA thermometers, e.g. conformational changes in RNA are used to modify transcriptional activity [2, 3]. The fluidity and thickness of membranes change with temperature, modifying the activity of transmembrane proteins, as it was shown for the histidine kinase DesK in bacteria [4]. In eucaryotes, members of the transient receptor potential (TRP) family of cation channels are prominent candidates for cold and heat sensing [5]. ThermoTRPs are expressed in several cell types and act by inducing membrane depolymerization and increasing intracellular calcium.

Another field of research is concerned with the question of how cells adapt their physiological processes to the environmental temperature change. Upon exposure of cells to elevated temperature beyond the physiological range, a heat shock response is induced that leads to an upregulation of molecular chaperones such as heat shock proteins, e.g. Hsp70 [6], or the formation of stress granules [7] to protect the proteome and stall translational activity [8, 9, 10]. Thereby, an interesting question is if temperature gradients, e.g. originating from metabolic activity, exist within cells. This topic was controversially discussed within the past 10 years after initial studies proposed that mitochondrial activity leads to a local temperature increase in the cell by 1 K as shown experimentally in luminescence nanothermometry [11, 12]. Calculations, by Baffou *et al*. [13], however found that the maximum temperature increase is five orders of magnitudes smaller, in the range of 10^−5^ K. This discrepancy was termed the ‘10^5^ gap’ [13, 14, 15, 16, 17, 18]. Possible temperature gradients within cells certainly have major implications since all molecular processes are temperature‐dependent. For example, enzyme activity may vary across a temperature gradient [19, 20]. Thermophoresis could lead to a different molecular distribution [21] and diffusion‐limited processes in signal transduction could be subject to thermal gradients [22].

These examples illustrate that in live‐cell imaging a precise control of temperature is needed. Commonly incubation chambers (either environmental boxes around the microscope or stage‐top incubators) are used, which also ensure a certain humidity and CO_2_‐content for a stable pH. Elevated temperatures can be used to induce cellular processes like chromosome separation in yeasts during meiosis using temperature‐sensitive alleles [23], tubulin repolymerization [24] or to investigate the nature of P granule assembly in *Caenorhabditis elegans* [25]. A rapid modulation of temperature within minutes and even seconds can be achieved by more advanced Peltier elements or microfluidic devices. Finally, heating by laser radiation or localized heat absorbers can be used to modulate biological processes with subcellular resolution similar to optogenetic approaches [26].

In this article, we first review techniques to monitor temperature in cells with microscopic resolution using optical thermometry (Fig. 1A). Secondly, we give a brief overview using advanced stage heating methods like Peltier elements and microfluidic devices. Third, we introduce (sub‐)cellular heating experiments using infrared (IR) laser radiation leading to a label‐free analysis of biomolecular composition of the cell (Fig. 1B), and furthermore to insights in cytoplasmic flow, protein activity, biomolecular stability and folding or interaction kinetics (Fig. 1C). Third, the benefit of local heat application via optical or magnetic excitation of nanostructures is discussed showcasing an example of induced transcriptional activity (Fig. 1D, left). Further examples include the modulation of Ca^2+^ signaling by controlling the flux of exogenous and endosomal calcium in the cytosol using magnetic radiation (Fig. 1D, middle) and controlled cell death by targeting specific cells with ultrasound radiation (Fig. 1D, right).

**Fig. 1:**
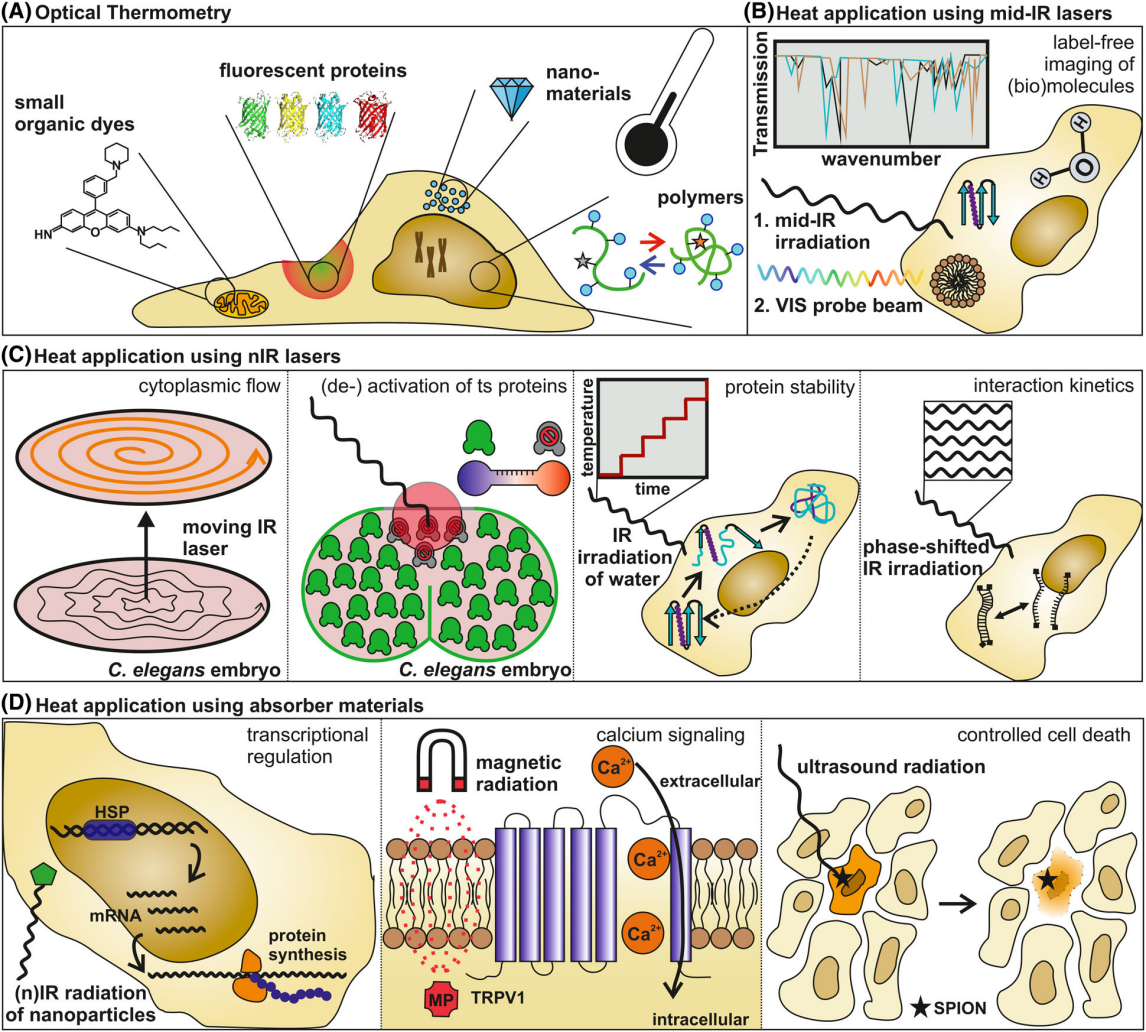
Thermal heating in live‐cell imaging to induce biological processes with high spatiotemporal resolution. (A) Optothermal sensors allow to monitor the temperature in the cell with microscopic resolution. Small organic dyes track different cellular compartments by temperature‐dependent fluorescence. Polymeric temperature sensors rely on conformational changes. Nanomaterials allow to engineer optical thermometers with versatile optical properties. Fluorescent proteins can be readily expressed in cells. (B) Direct heating with mid‐IR radiation allows label‐free detection of (bio)molecules. (C) Direct heating of cells by nIR radiation is used to induce cytoplasmic flow, inactivate temperature‐sensitive (ts) proteins, probe protein stability and sample interaction kinetics inside cells. (D) Indirect heating via excitation of nanostructures using optical, magnetical or ultrasound excitation. Examples include the optical excitation of nanoparticles to regulate transcriptional activity utilizing a heat shock promoter (left), magnetic excitation of magnetic particles (MP) controlling calcium signaling (middle), and ultrasound excitation of nanoparticles for controlled cell death in cancer treatment (right).

## Optical thermometry

In most applications, precise temperature measurements inside cells are a prerequisite that can only be achieved using molecular thermometers, mostly coupled to an optical readout such as fluorescence intensity, polarization, or lifetime. The so‐called optical thermometers can be used for two different purposes: They report on temperature changes induced by extrinsic heating sources such as IR lasers [27] or on intrinsic temperature changes within cellular systems e.g. in the nucleus vs. cytosol [11] or the cell body vs. neurite outgrowth [28]. They can also be used to monitor temperature gradients possibly originating from biological processes like cell cycle [29] or mitochondrial activity [30].

**Fig. 2:**
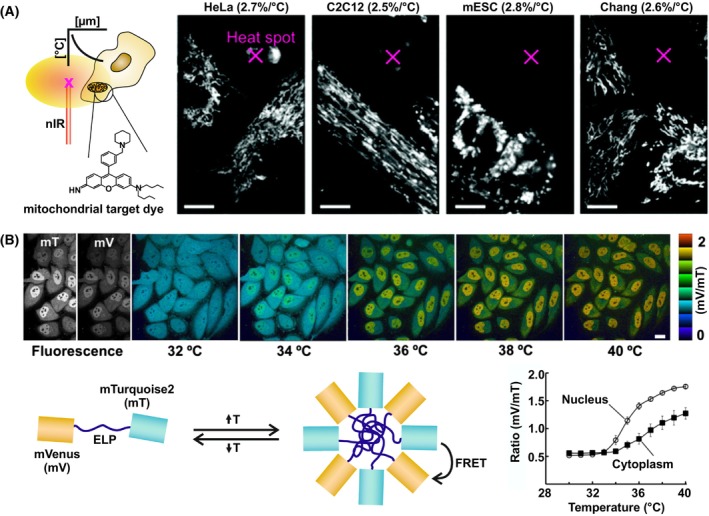
Optical thermometers to determine intracellular temperatures. (A) A rosamine‐based fluorescent dye colocalized with mitochondria serving as an organelle specific temperature probe. Aluminum particles were heated with a nIR laser in proximity to the cells. The images show its application in different cell cultures with a comparable temperature sensitivity of 2.5–2.8% per °C. Scale bar = 20 μm. Adapted and reprinted from [31] with permission. (B) Genetically encoded temperature sensor based on the fluorescent proteins mVenus (mV) and the mTurquoise2 (mT) separated by elastin‐like polypeptide linker (ELP). Upon temperature increase the sensor self‐associated, allowing a ratiometric readout of the temperature (mV/mT). The study revealed a temperature difference between the cytosol and the nucleus in HeLa cells. Scale bar = 20 μm. Adapted and reprinted from [34] with permission.

A broad range of molecules and materials was tested for this purpose (Fig. 1A): (a) Small organic dyes are readily available with high specificity for different cellular organelles (Fig. 2A) [31]. (b) Tailored polymers allow cellular uptake and a homogenous distribution throughout cells and even the entire cell organism [32, 33]. Conformational transitions provide a more robust readout to environmental changes compared to the fluorescence of small organic dyes [34]. (c) Engineered nanomaterial feature high photostability and robustness, rendering them suitable for long‐term cell tracking [35], application in super‐resolution microscopy [35] and nanoscale temperature sensing [36]. (d) Transfection of genetically encoded sensors based on the fluorescent protein GFP allows for noninvasive provision of the optical thermometer. Thereby, experiments in specific subcellular compartments [37] but also entire organisms are possible (Fig. 2B) [34, 38].

The major challenge in the development and application of optical thermometers is that they need to be insensitive to environmental changes such as pH, ionic strength, osmolyte concentration or crowding. Such properties may vary substantially within cells or between individual cells of a culture [39]. Thus, the accuracy and reliability of optical thermometers in different applications is still unclear. This is illustrated by the previously mentioned discussion of the ‘10^5^ gap’ referring to the difference between a predicted temperature gradient between mitochondria and cytosol [13] and experimental values by optical thermometry [11, 14, 15, 16, 17, 40, 41]. For a more comprehensive review of optical thermometers the reader is referred to a recently published book by Uchiyama [42].

### Heat application using stage heating

Environmental boxes and stage‐top incubators enable simultaneously a constant temperature and CO_2_ control to ensure stable conditions in live‐cell imaging. Due to the size of the incubators, temperature modulation to investigate dynamic processes in the cell is hardly applicable. Stage‐top incubators based on Peltier elements and microfluidic devices overcome these temporal limitations and allow homogenous heating of the whole sample carrier. To study the cell cycle in yeast, a microfluidic device was used to arrest a temperature‐sensitive yeast strain in the G2 phase of the cell cycle by incubating the yeast cells at a restrictive temperature of 36.5 °C. A rapid temperature shift (< 10 s) to the permissive temperature of 25 °C allows then a synchronous cell cycle re‐entry [43]. On a whole‐organism‐level a Peltier element was used to modulate P granule formation in *C. elegans*. P granules are RNA‐rich condensates, that assemble via phase separation. Dissolution of the P granules with increasing temperature and recondensation upon lowering the temperature could be investigated, obtaining in‐cell phase diagrams [25]. For these heating techniques sample size can be further scaled up: Ahl *et al*. [44], designed a microfluidic device that allows temperature control of whole tissues. Leukocyte migration and local blood flow were investigated cycling between healthy (37 °C) and heat conditions (42 °C) in tissue, displaying a natural environment. In summary, stage‐top incubators are a robust tool to maintain temperatures constantly and also allow dynamic temperature modulation on a second to minute timescale, observing individual cells with high spatial resolution as well as a population of cells, organisms or even whole tissues.

### Heat application using IR lasers

IR lasers are used to directly heat cellular water in living cells. Diode lasers of different wavelengths in the near and mid‐infrared can be used matching the broad absorption of water in this frequency range [45]. The laser can be applied from the top of an inverted fluorescence microscope or, alternatively, through the optical beam path using IR compatible optics. A laser power of 1 W is sufficient to heat adherent mammalian cells (e.g. in an area of 100 × 100 μm in a 300 μm thick sample chamber) uniformly within milliseconds. Alternatively, the laser can be focused down to a few micrometers (diffraction limit) for spot heating. Different techniques were developed that apply IR laser heating in combination with live‐cell imaging.

MIP (mid‐infrared photothermal imaging) enables label‐free visualization of cellular components and biomolecules that overcomes the limitations of the vibrational mid‐IR microscopy, including high scattering, low spatial resolution and high water absorption [46]. In MIP a mid‐IR laser is used as a pump beam for vibrational excitation. Molecular absorption of mid‐IR light induces local heating and therefore a change in the refractive index. Subsequently, the change in refractive index is probed with a VIS‐laser. Alterations in the refractive index result in changes of phase, reflectance or scattering intensity of the probe beam. Therefore, spatial distribution of mid‐IR absorption is obtained with sub‐μm resolution. Vibrational modes of amides, sp^2^ and sp^3^ hybridized CH_x_ and OH‐bonds allow to distinguish between basic components of the cell like proteins, lipids and water, respectively [47]. In one application, MIP was used to investigate the function of aquaporines by observing the flux of D_2_O across the membrane [47]. In two other studies, a metabolic analysis on a single bacterium level [48] or in adipocytes was performed by imaging carbohydrate conversion into biomass and *de novo* lipogenesis, respectively [49]. Further improvement, of either mid‐IR excitation using an optical parametric oscillator [47] or signal detection, enhancing the signal with wideband voltage amplifier and a megahertz digitizer [48], allows to increase imaging speed by two orders of magnitude. In summary, MIP allows label‐free imaging in cells whole organisms of protein dynamics [50] and cellular response to antibiotics [51], and biomolecular mapping [52] with high spatiotemporal resolution.

**Fig. 3:**
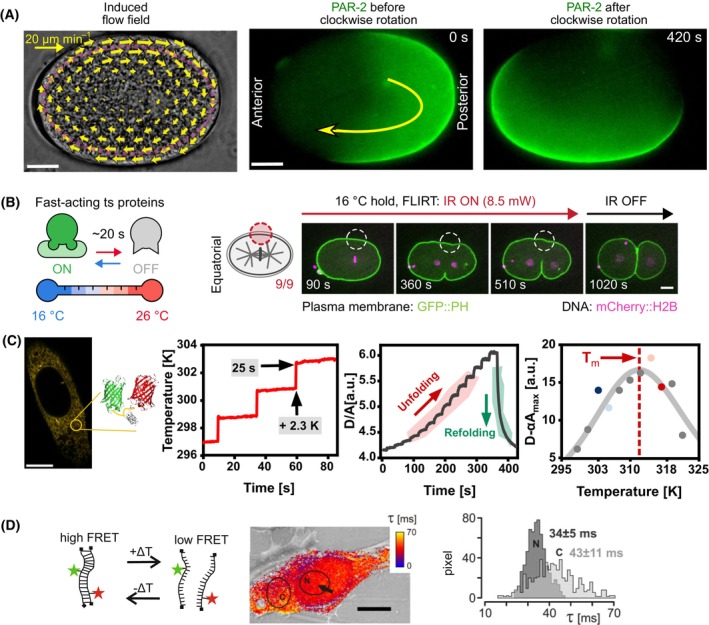
Heat application by direct excitation using IR lasers. (A) FLUCS was used to induce a cytoplasmic flow by clockwise movement of an IR laser in a *C. elegans* zygote. The pattern of the GFP‐tagged partition‐defective protein (PAR) was redistributed 420 s after the induced flow. Scale bar = 10 μm. Reprinted from [53] with permission. (B) FLIRT allowed reversible inactivation of temperature‐sensitive proteins (myosin‐II) in *C. elegans* embryos during cell division using spatially controlled IR laser heating. Scale bar = 10 μm. Reprinted from [58] with permission. (C) Thermal stability and unfolding of superoxide dismutase 1 (SOD1) in the endoplasmatic reticulum investigated by FReI. SOD1 was labeled C‐ and N‐terminally with a FRET pair to track unfolding. A temperature profile was applied to the cell to induce stepwise unfolding. Kinetic unfolding amplitudes were used to determine the melting temperature (*T*_m_) as a measure of folding stability. Scale bar = 20 μm. Reprinted from [74] with permission. (D) TOOL microscopy was used to study DNA hybridization by an oscillating temperature. Pixel‐by‐pixel analysis revealed millisecond kinetics within single cells. Scale bar = 10 μm. Reprinted from [73] with permission.

FLUCS (focused light‐induced cytoplasmic streaming) was used to investigate physical transport processes inside the cell such as diffusion or cytoplasmic flow [53]. An IR laser was used for spot heating and its spatial movement creates a thermoviscous intracellular flow (Fig. 3A). As an example, the morphogenesis of *C. elegans* was investigated by FLUCS. The central event at the onset of *C. elegans* development is the polarization of the zygote prior to its asymmetric division into differently sized daughter cells. This polarization is mediated by the localization of partitioning defective proteins (PARs) at opposite poles. By inducing a directed flow towards the posterior pole, increased PAR‐2 loading onto the posterior membrane was achieved (Fig. 3A). Additionally, already membrane‐bound PAR proteins could be translocated along the membrane. A combination with flow‐induced movement of the entire actomyosin cortex showed that the physical flows of the actomyosin cortex alone were sufficient to relocate PAR domains along the membrane. Furthermore, FLUCS was used to investigate cell motility [54] or intracellular trafficking [55] and to measure the rheological properties of the nucleus and its sub‐compartments revealing insights into nuclear organization [56]. *In vitro*, FLUCS was used to actively manipulate micrometer‐sized objects, bringing biomolecular condensates into proximity [57]. In summary, FLUCS allows to probe intracellular transport processes on the micrometer scale in a probe‐ and contact‐free mode and can thereby be used for the directed movement of cellular architecture.

FLIRT (fast local IR thermogenetics) was introduced as a method to control temperature‐sensitive proteins with subcellular precision [58]. The spatial heating profile is controlled by an IR laser focused through the beam path of a spinning disk confocal microscope. An additional optical mask was used in the IR beam path to induce spatially controlled heating profiles. Using this capacity, Hirsch *et al*. showed in one‐cell *C. elegans* embryos that cell division was impaired by thermal inactivation of equatorially located myosin‐II. By a locally constricted heating of the embryonal cell, ring constriction was inhibited at the respective spot and was only observed on the opposite equatorial side (Fig. 3B). The temperature‐sensitive mutant of myosin‐II reacts to temperature shifts from 16 °C to 26 °C which is well in the range of the physiological maintenance temperature of *C. elegans* [59]. Ring constriction was completed upon laser deactivation and recooling, showing reversibility of the manipulation. Importantly, the treatment neither disrupted polarity or asymmetry of cell division nor embryo viability or development timing. In another experiment, FLIRT was also applied to control cell‐cycle timing in *C. elegans* embryos, providing insights into timing regulation mechanisms during early embryogenesis [60]. FLUCS and FLIRT are both techniques that allow to combine high‐resolution confocal imaging with a high precision control of sample heating using focused or spatially shaped infrared laser spots.

In contrast, in Fast Relaxation Imaging (FReI) the entire field of view of the microscope is heated homogeneously by an IR laser but temporally modulated in intensity. This allows to control the sample temperature with time (< 1 ms) and to adjust the heating profile to produce a temperature jump. Thereby, temperature relaxation experiments, as originally developed by Manfred Eigen to study chemical kinetics [61], can be conducted on a microscope stage. FReI was used to investigate biomolecular folding and stability *in vitro* and *in vivo* using stepwise 2–4 °C temperature jumps in combination with intramolecular Förster resonance energy transfer (FRET) (Fig. 3C) [62]. For example, FReI was applied to study the unfolding and aggregation of disease‐related proteins, like PAPS synthase 2 (PAPPS2) [63, 64] or superoxide dismutase 1 (SOD1) [65, 66] in living cells. Similarly, FReI can be used to study nucleic acids such as CAG‐repeat RNAs [67, 68]. The experiments allowed to access biomolecular folding and aggregation landscapes directly *in cellulo* and *in vivo* (zebra fish) yielding novel insights into cellular factors that influence these pathways like cellular stresses [69], macromolecular crowding [69, 70] or phase separation [71, 72].

In a similar approach, temperature oscillation optical lock‐in (TOOL) microscopy uses IR laser‐induced temperature oscillation to perturb the biomolecular system and to retrieve kinetic and thermodynamic information. Compared to FReI, that allows to analyze kinetics occurring on millisecond to second timescales, TOOL microscopy covers micro‐ to milliseconds by varying the periodic heating frequency in combination with lock‐in detection. In an application, Schoen *et al*. [73] used the method to investigate hybridization kinetics of double‐stranded DNA (Fig. 3D) *in vitro* and in cells. They found that hybridization kinetics *in vitro* were independent of the number of base pairs, while hybridization kinetics in the cell were dependent on DNA binding partners, preferring nucleic acids of a certain length. Although the technique was not widely used yet, it offers a versatile platform to investigate biomolecular reactions on fast timescales.

The methods FLUCS, FLIRT, FReI and TOOL show that direct IR laser heating of the specimen in live‐cell imaging is a powerful technique to probe and manipulate cellular processes.

## Heat application using absorber materials

Thermal modulation of biological samples can also be achieved by indirect heating using different nanomaterials as absorbers. Based on the nature of the nanoparticle, excitation can be achieved by optical irradiation or in a magnetic field. The resonant optical excitation of electrons in semiconductors and metals, as well as excitation of magnetic particles in an alternating magnetic field, is converted into heat release from the particle. Therefore, the use of nanomaterials allows heating with a high spatial accuracy due to their nano‐ to micrometer size. This precision cannot be accomplished by direct heating using focused IR lasers (focal diameters typically > 2 μm).

Ferdinandus *et al*. [75] used an optical polymeric nanoparticle to investigate muscle contraction in C2C12 myotubes. The polymeric nanoparticle was composed of a fluorescent thermometer dye and a photothermal dye embedded in a polymer matrix. This so‐called nanoheater‐thermometer (nanoHT) allowed thermal modulation and a direct readout of the temperature at the same time. A subcellular‐sized heat spot (5 μm) was generated by the illumination of single nanoHT with a 808 nm nIR laser, that altered actin‐myosin interaction in a limited area of the cell leading to locally restricted contraction. With this approach Ferdinandus *et al*. even surpassed the spatial resolution of preceded studies, that showed muscle contraction of whole cells by either direct heating of the cellular environment [76] or indirect heating with a number of internalized gold nanoshells [77]. In summary, the method allowed for subcellular heating approaches with unprecedented spatial resolution of < 8 μm [75].

**Fig. 4:**
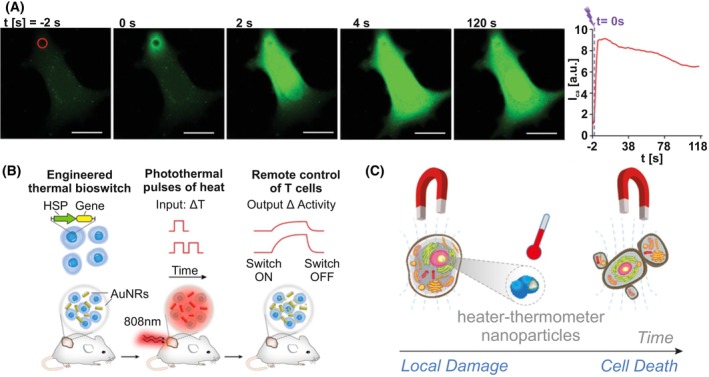
Indirect heat application by opto‐ and magnetothermal nanoparticles. (A) Calcium release from endosomes was induced by exciting plasmonic nanoparticles close to endosomes. Ca^2+^ release and distribution in the cell was monitored by Fluo‐4AM. Scale bar = 20 μm. Reprinted from [78] with permission. (B) Gene expression was triggered by activation of thermal gene switches which are based on the promoter of the heat shock protein HSP70B'. Pulsed excitation of gold nanorods enabled long‐term control of transcriptional activity in engineered T‐cells in mice. Reprinted from [79] with permission. (C) Magnetic nanoheaters were used to induce local hyperthermia and therefore control the cell death of particular cells. Reprinted from [89] with permission.

In another example polymer capsules with integrated plasmonic nanoparticles were used to modulate Ca^2+^‐signaling, which is an important pathway of intra‐ and intercellular communication. Therefore, cells were loaded with the nanoparticles, that reside in the endo lysosome [78]. These micrometer‐sized capsules were individually heated resulting in the release of calcium from the lysosomes, increasing cytosolic Ca^2+^ levels (Fig. 4A). This approach is independent from the influx of exogenous Ca^2+^ through the transient receptor potential vanilloid 1 (TRPV1), which can also be heat‐activated, as it is described below by magneto thermal manipulation. Therefore, modulation of intracellular calcium levels can also be investigated in TRPV1‐negative cells. Alteration of intracellular calcium concentrations allows e.g. to control Ca^2+^‐mediated gene expression with Ca^2+^‐sensitive promoters, or in drug screening to test compounds interfering with Ca^2+^ spreading via gap junctions.

In a last example, gene expression was directly modulated by increased temperatures. Thermal gene switches were constructed from the heat shock protein HSP70B' (HSPA6) promoter which has basal transcriptional activity and activates within an elevated temperature window of 40–42 °C. Abedi *et al*. showed, that local plasmonic photothermal heating of gold nanorods with an IR laser allow spatial and remote control of transcriptional activity and is applicable *in cellulo*. Furthermore, it was tested in implants in mice to specifically regulate T‐cells (Fig. 4B) [79]. Thermal gene switches offer a great opportunity for precise gene regulation, so that it was used e.g. for the control of CAR T‐cell activity in tumor defense [80]. In principle, any kind of gene expression can be regulated: in another study, dCas9‐mediated transcriptional activity was controlled by thermal gene switches [81], enabling a multiplexed approach to remotely interrogate mammalian biology.

Heat application can also be realized using ultrasound waves in combination with superparamagnetic iron oxide nanoparticles (SPIONS). High‐intensity focused ultrasound heating (HIFU) is achieved using an ultrasound generator and a piezoelectric transducer: In general, acoustic energy (1–7 MHz) is generated and detected using piezoelectric crystals. In an application, ultrasound waves were focused on a focal area in which the energy was converted to thermal energy within seconds. The additional use of SPIONs allows to reduce acoustic energy to ≤ 1 MHz and ensure local heating of cancer cells [82, 83]. Heat application with ultrasound radiation without local conversion by nanoparticles and high acoustic energy was used for the treatment of breast [84], kidney and liver [85], and prostate cancer [86], however healthy tissue was commonly also affected [87, 88]. Therefore, the combination of HIFU and SPIONs offer the advantage to reach target regions with high penetration depth and long application time to induce local cell death and reduced impairment of surrounding cells.

Alternatively, cancer cells can be targeted using magnetic fields to excite magnetic nanoparticles in the respective cells (Fig. 4C) [89]. The applied heater/thermometer nanostructures allowed for the generation of a local temperature increment within minutes of 5.9 °C in vicinity of the particles. This temperature increase was sufficient to induce cell death after 24 h and was generated by applying a moderate magnetic field for 30 min, which is still well within the healthy limit values [89]. In a neurochemical context, Rosenfeld *et al*. made use of this technique to control the transient receptor potential channel family (TRPV1,3 and 4) and therefore the flux of exogenous cations across the plasma membrane of neuronal cells. This led ultimately to the firing of action potentials [90, 91] and also to neurite elongation upon magnetothermal stimulation and increased migration of Schwann cells [92]. Magnetic nanoparticles can also be applied *in vivo*. Using *C. elegans* nematodes it was shown that local heating via magnetic nanoparticles of sensory neurons inhibited their forward locomotion and initiated backward locomotion showing a thermal avoidance reaction [90]. Furthermore, neurons of the reward system in the brain of mice were first transfected to overexpress TRPV1 and secondly injected with magnetic nanoparticles to induce neuronal excitation in a magnetic field [91].

In summary, nanomaterials are versatile building blocks for the design of nanoheaters. They can be specifically tailored to match the needs of the application in terms of localization and excitation source. They ensure precise spatial heating due to their nanometer size, rendering them suitable for manipulating and analyzing subcellular processes.

## Conclusion

Most biomolecular processes are highly temperature‐dependent and only operational in a narrow range. Temperature must be tightly controlled in live‐cell imaging applications; however, controlled changes in temperature serve as a powerful tool to manipulate and probe cellular processes. We here presented different methods for heat application in live‐cell imaging, each tailored for specific applications. A summary of the different techniques together with their respective applications and experimental challenges is given in Table 1.

**Table 1:** Overview of heat application techniques in live‐cell imaging.

Method	Application example (s)	Special features	Challenges	Heating resolution temporal/spatial	Applicable imaging techniques	Applied temperature sensor (s)	References
Microfluidics, Peltier elements	Liquid–liquid phase separation, Cell‐cycle control	Homogenous heating of large samples e.g. tissue	Low temporal resolution	s‐min/whole sample carrier	Widefield fluorescence microscopy	Thermal camera (tissue), resistance temperature detector	[25, 43]
MIP Mid‐infrared photothermal imaging	Protein dynamics, Biomolecular mapping	Label‐free	Spatial resolution limited by probe beam; Advanced excitation and detection for high temporal resolution	20 ms/5.5 μm	Widefield fluorescence microscopy	mid‐IR signal (measured as modulated probe power)	[46, 47]
FLUCS Focused light‐induced cytoplasmic streaming	Advective transport in cells	Dynamic manipulation of cellular processes	Precise temporal control of IR laser focus and position	300 ms/10 μm	Widefield fluorescence microscopy	Rhodamine B	[53, 56, 57]
FLIRT Fast local IR thermogenetics	Switching of fast‐acting temperature‐sensitive proteins	High spatial resolution in heat application	Precise spatial control of IR laser focus and position; Mutational studies required to create fast‐acting temperature‐sensitive proteins	20 s (depending on temperature‐sensitive protein)/16–27 μm	Spinning disc confocal fluorescence microscopy	mCherry::Histone2B in *C. elegans*	[58, 60]
FReI Fast relaxation imaging	Biomolecular folding stability and kinetics in living cells	High temporal resolution (ms‐min time scale)	Precise temporal control of IR laser intensity; Temperature‐dependent studies limited by heat tolerance of cells	< ms/1 mm	Widefield fluorescence microscopy	Rhodamine B, mCherry	[62, 64, 65, 97, 98]
TOOL Temperature oscillation optical lock‐in microscopy	Biomolecular association kinetics	High temporal resolution (μs‐s time scale)	Modulation of IR intensity and lock‐in detection required; Temperature‐dependent studies limited by heat tolerance of cells	μs‐s/field of view	Confocal fluorescence microscopy	Cy‐5‐labeled DNA	[73]
Optical nanoheater	Transcriptional activity; Calcium signaling; Muscle contraction (protein–protein interaction)	Compatibility with light microscopes; Broad variety of tunable nanoparticles	Biocompatibility and cytotoxicity of nanomaterials; Delivery to cells and tissue	5 s/5 μm, e.g. for [75] in general dependent on nanoparticle size	Widefield and confocal fluorescence microscopy	EuDT/C102 (with Metallo‐phthalcyanines as nanoHT) [75]	[75, 77, 78, 79]
Magnetic nanoheater	Calcium signaling; Controlled cell death in cancer treatment	High penetration depth into tissue	Combination of magnetic fields and microscopy; Biocompatibility and cytotoxicity of nanomaterials	s‐min/heating: field of view [90]	Widefield and confocal fluorescence microscopy	DyLight549, YFP, ANNINE6 [90]	[89, 90, 91, 99]

In the first group of techniques advanced stage heating methods (Peltier elements and microfluidic devices) are presented allowing for temperature changes on the timescale of seconds to minutes. Although these heating techniques lack a high temporal resolution, large sample specimens or multiwell plates can be heated homogenously and heating devices are easily implemented in the microscopic setup. In the second group of techniques (FLUCS, FLIRT, FreI, and TOOL) direct optothermal heating is achieved by exciting the vibrational band of cellular water using IR lasers. The advantage is that heat modulation can be performed without considering delivery, biocompatibility and cytotoxicity of nanoparticles. Heat application with the highest spatial resolution (diffraction limited) is achieved in FLIRT, using μm‐diameter masks for IR excitation and therefore, allowing for subcellular heating and heat‐induced manipulation of cellular processes. In FLUCS a high spatial but also temporal control of the moving laser beam is required to induce a cytoplasmic flow either on a single cell level or on a subcellular level to probe transport processes. FReI and TOOL use temperature modulation to probe biomolecular stability and dynamics in cells in comparison to test tube conditions. In both applications the whole field of view is heated, to ensure homogenous heating of entire cells. Still, the methods protrude with their temporal resolution: While FReI allows to access ms to min timescales, TOOL operates from μs to s. Both techniques are limited by the heat tolerance of the cell or organism under investigation. In the above‐mentioned methods, IR radiation is used to excite water molecules, producing heat in turn. In MIP mid‐IR radiation is used to monitor the (bio)‐molecular composition of the cell. Excitation of biomolecules is complemented by a readout with a VIS‐laser allowing higher spatial resolution compared to mere mid‐IR microscopy. The strength of MIP lies in the label‐free detection of molecules, only relying on the vibrational excitation of different bonds.

In the third group of methods, nanoheaters are used for indirect heat application with the highest spatial resolution (up to 100 nm), allowing to control localized cellular processes such as transcriptional activity or calcium signaling. Compared to the direct heating methods, sophisticated optics/techniques to spatio‐temporally control the IR laser spot are not required. However, the targeted delivery of nanomaterials to cells and tissue as well as their biocompatibility and cytotoxicity must be considered in their application. Each of the excitation techniques comes along with certain advantages and challenges. The advantage of optical excitation over magnetical and ultrasound excitation is, that the laser beam can easily be focused on individual cells. Conversely, magnetic and ultrasound excitation are always applied to several cells, since cells are placed in a magnetic coil and ultrasound is often applied in tissue. This rises the need to keep excitation power low to avoid damage of non‐targeted cells. Still, this awareness enables a high penetration depth for magnetical and ultrasound excitation while application of optical excitation is limited by high absorption and scattering in thick samples. The use of magnetic nanoheaters covers a broad range of temporal resolution, ranging from seconds, to very locally induce heat changes in neuronal application, to a timescale of several minutes, to induce targeted cell death. In contrast to that HIFU is mainly applied to induce controlled death of targeted cells and therefore, by now, only dealing with timescales of several minutes. Optical excitation allows fast heating on a second range, making it suitable to investigate dynamic processes of biomolecular interactions. The large variety of different nanoheaters (e.g. excitation source, intra‐ or extracellular application, subcellular targeting, spatial and temporal resolution…) allow the selection of a tailored candidate for the respective application.

While stage heating is widely used in live‐cell imaging, it is expected that local and fast heating techniques using direct or indirect methods will lead to exciting applications in cell biology in the future. The field of biomolecular condensates is an example in which this capacity is required. Fast and local heating can be applied to induce phase separation and record intracellular temperature‐dependent phase diagrams. This will allow to study the nature and function of biomolecular condensates in cells [25]. Heat application could also be used to induce aberrant phase transitions and aggregation processes associated with various neurodegenerative diseases [93, 94]. An example is the aggregation of a mutant Huntingtin exon‐1 protein (involved in the onset of Chore Huntington) which can be induced by IR laser heating [95, 96]. Such applications will vice versa trigger novel technical developments for heat application in live‐cell imaging including nanoheating and thermosensing technology.

## Conflict of interest

The authors declare no conflict of interest.

## Author contributions

L.S. planned and wrote the manuscript. S.E. wrote and proofread the manuscript.
